# Modeling Spatial Patterns of Traffic-Related Air Pollutants in Complex Urban Terrain

**DOI:** 10.1289/ehp.1002519

**Published:** 2011-01-24

**Authors:** Leonard M. Zwack, Christopher J. Paciorek, John D. Spengler, Jonathan I. Levy

**Affiliations:** 1 Department of Environmental Health and; 2 Department of Biostatistics, Harvard School of Public Health, Boston, Massachusetts, USA; 3 Department of Statistics, University of California–Berkeley, Berkeley, California, USA; 4 Department of Environmental Health, Boston University School of Public Health, Boston, Massachusetts, USA

**Keywords:** mobile measurements, mobile sources, regression, spatial variation, ultrafine particles, urban air quality

## Abstract

**Background:**

The relationship between traffic emissions and mobile-source air pollutant concentrations is highly variable over space and time and therefore difficult to model accurately, especially in urban settings with complex terrain. Regression-based approaches using continuous real-time mobile measurements may be able to characterize spatiotemporal variability in traffic-related pollutant concentrations but require methods to incorporate temporally varying meteorology and source strength in a physically interpretable fashion.

**Objective:**

We developed a statistical model to assess the joint impact of both meteorology and traffic on measured concentrations of mobile-source air pollutants over space and time.

**Methods:**

In this study, traffic-related air pollutants were continuously measured in the Williamsburg neighborhood of Brooklyn, New York (USA), which is affected by traffic on a large bridge and major highway. One-minute average concentrations of ultrafine particulate matter (UFP), fine particulate matter [≤ 2.5 μm in aerodynamic diameter (PM_2.5_)], and particle-bound polycyclic aromatic hydrocarbons were measured using a mobile-monitoring protocol. Regression modeling approaches to quantify the influence of meteorology, traffic volume, and proximity to major roadways on pollutant concentrations were used. These models incorporated techniques to capture spatial variability, long- and short-term temporal trends, and multiple sources.

**Results:**

We observed spatial heterogeneity of both UFP and PM_2.5_ concentrations. A variety of statistical methods consistently found a 15–20% decrease in UFP concentrations within the first 100 m from each of the two major roadways. For PM_2.5_, temporal variability dominated spatial variability, but we observed a consistent linear decrease in concentrations from the roadways.

**Conclusions:**

The combination of mobile monitoring and regression analysis was able to quantify local source contributions relative to background while accounting for physically interpretable parameters. Our results provide insight into urban exposure gradients.

Epidemiological studies have shown a link between traffic-related pollutants or traffic levels and adverse health effects including coronary heart disease and respiratory symptoms (Janssen et al. 2003; Kim et al. 2004; Rosenlund et al. 2008). These studies have led to interest in the concept of “hot spots,” which are locations where mobile sources may expose people to elevated levels of air pollutants (Zhou and Levy 2007). Hot spots are challenging to characterize, because regulatory monitors are designed and sited to monitor compliance with air-quality standards and are not ideally equipped to assess or capture local hot spot formation arising from mobile sources.

A number of field studies have shown increased traffic-related air pollutant concentrations near major roadways with heavy traffic (Kaur et al. 2005; Weijers et al. 2004; Zhu et al. 2002). However, these studies did not generally include spatial characterization beyond a linear transect or formally incorporate predictors of pollutant concentrations—steps that are needed to develop interpretable characterizations of hot spots. Moreover, most did not include settings with complex urban terrain or multiple large roadways in close proximity, a common occurrence in urban areas.

Some recent monitoring studies have addressed predictors beyond proximity to major roadways. In a recent review article, Kaur et al. (2007) concluded that mobile-monitoring studies rarely formally incorporate traffic and meteorological parameters that are associated with concentrations of ultrafine particulate matter (UFP) and fine particulate matter [≤ 2.5 μm in aerodynamic diameter (PM_2.5_)]. Recent mobile-monitoring studies have reinforced these findings. A study conducted in Montreal, Canada, found UFP concentrations measured in various transportation microenvironments to be inversely associated with temperature and wind speed (Weichenthal et al. 2008) but did not investigate the impacts of traffic. Another study conducted in Beijing, China, found a strong impact of traffic on UFP concentrations but did not formally incorporate meteorological parameters (Westerdahl et al. 2009). In a study in Boston, Massachusetts (USA), that characterized spatial patterns of UFPs and PM_2.5_ near two urban roadways, Buonocore et al. (2009) found significant distance-dependent relationships, but they had limited traffic data concurrent with mobile monitoring and did not find consistent associations with wind speed or direction. To our knowledge, no mobile-monitoring studies have incorporated the key dimensions known to be important, based on atmospheric dispersion principles, into a single model. Thus, there is a need for a statistical model that can assess the joint impact of both meteorology and traffic on measured concentrations of mobile-source air pollutants over space and time.

To determine the influence of local sources as well as meteorology and distance to roadway on spatial distributions of mobile-source air pollutants in an area with complex urban terrain, we chose a sampling area in the Williamsburg neighborhood of Brooklyn, New York (USA). The Williamsburg neighborhood is affected by two major sources of traffic-related air pollution: the Williamsburg Bridge (WB) and the Brooklyn–Queens Expressway (BQE). These two sources have complex geometries because they are often elevated or below ground level, which can lead to the formation of pollution hot spots that may be difficult to characterize using traditional methods. Our hypothesis is that continuous characterization of traffic, local meteorology, and other statistical parameters will allow for quantification and characterization of distance-dependent relationships relative to background concentrations for mobile-monitored concentrations of UFPs and PM_2.5_.

## Materials and Methods

### Study area and monitoring equipment

**Figure 1:**
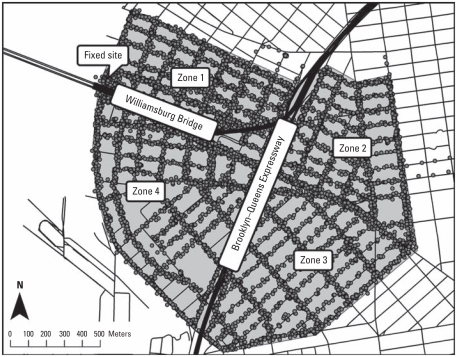
Map of Williamsburg sampling zones. Each dot represents the location of a 1-min–averaged sample. Fixed site refers to the location of the weather station. Zones 1–4 refer to the four predesignated sampling zones.

In June 2007, a 3-week sampling campaign was conducted in the Williamsburg section of Brooklyn, New York, as part of the New York Metropolitan Exposure to Traffic Study. Figure 1 is a map of the area, which included four sampling zones.

Three sampling backpacks were created containing instrumentation that could measure 1-min–averaged concentrations of UFPs, PM_2.5_, and polycyclic aromatic hydrocarbons (PAHs). Over the course of the study, six different people carried the backpacks. For UFPs, water-based condensation particle counters were used (model 3781; TSI Inc., Minneapolis, MN), which can detect particles as small as 6 nm (TSI Inc. 2007). Two types of aerosol monitors—the TSI DustTrak 8520 and the EcoChem PAS2000CE (EcoChem Analytics, League City, TX)—were used to sample PM_2.5_ and PAHs, respectively. In addition to the pollution-monitoring instruments, each backpack was outfitted with a Garmin GPSMAP 60CSx global positioning system device (Garmin International, Inc., Olathe, KS), to continuously record the spatial location of the backpack as it moved through the study area, and a HOBO Pro data logger (ONSET Computer Corp., Bourne, MA) to record temperature and relative humidity (RH). A WeatherWizard III weather station (Davis Instruments Corp., Hayward, CA) was deployed on the roof of a three-story apartment building inside the sampling area to characterize wind speed and direction.

To capture traffic levels on the BQE and the WB, a consultant from American Traffic Information, Inc., Staten Island, New York, was hired to set up automated continuous traffic counters on the outer lane on each side of each roadway. The consultant set up a total of five traffic counters: Two were located on the WB and three were located on the BQE; only a subset of the counters were used in the final analyses. The traffic parameters collected included vehicle counts, Federal Highway Administration vehicle class, and vehicle speed. The traffic counts were measured continuously and provided 15-min counts.

For quality assurance and control, field staff involved in data collection received training on how to properly fill out all log sheets, how to use the sampling equipment, and how to calibrate equipment. Equipment was zero-balanced at the beginning of each sampling shift, and before the study, the pollution monitoring instruments had been factory calibrated. Measurements taken at the same time and location by different instruments before the field study demonstrated good correlations and sufficient performance for our analyses. When postprocessing the data, instrument logs and field logs were examined to ensure that there was no instrument or operator error, and less than 5% of the original samples were excluded because of abnormally low instrument readings or measurements that occurred outside of the sampling session.

### Sampling protocol

Each of the three backpacks was carried along scripted walking routes that we designed to ensure thorough spatial coverage of all roads in the area. To help limit the confounding of spatial and temporal effects, each of the four sampling zones had up to four different walking routes assigned to it, and the routes were randomized so that they were covered exhaustively at different times of day, during different days of the week. Each individual sampling shift lasted for approximately 2.5–3 hr, with two of these shifts occurring from ~ 0900 to 1200 hours and from ~ 1400 to 1700 hours per day.

### Statistical analysis

Data analysis was conducted using SAS (version 9.2; SAS Institute Inc., Cary, NC), ArcGIS (version 9; ESRI, Redlands, CA), and R (version 2.10.1; R Development Core Team, Vienna, Austria) along with version 1.6-1 of the *mgcv* package (Wood 2008). An alpha level of 0.05 was used to determine statistical significance throughout the study. We created spatial plots of the aggregate pollutant surfaces using a bivariate smooth of location for both UFPs and PAHs. For PM_2.5_, we created the pollutant surface plot using the same method described above while also accounting for temporal variation associated with local and regional sources with significant diurnal contributions. This temporal variation was accounted for by using a categorical variable that adjusts the spatial surface to account for the individual sampling shift that provided the measurements. These maps were created as a visual tool to assess average spatial patterns before adjusting for meteorology or other predictive variables.

Additive models depicting the effects of traffic, distance to nearest source, wind speed, temperature, and RH were created for each of the pollutants studied to assess the impacts of both the BQE and the WB simultaneously. Although wind direction would have a clear effect on concentrations in principle, our sampling occurred almost exclusively in low wind-speed conditions with variable wind directions, which can potentially lead to a spreading of the aerosol plume in multiple directions, including upwind (Hanna et al. 2003). We therefore constructed our primary models without a wind-direction term but considered the implications of this omission in our sensitivity analyses.

Our initial additive models were fitted using the gam function of the *mgcv* package and took the following form:


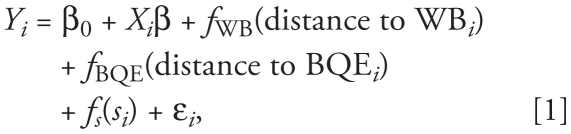


where *Y_i_* is the 1-min–averaged, log-transformed pollutant concentration; β represents the slope estimates for a matrix of covariates *X_i_*, which includes traffic count on the BQE, traffic count on the WB, wind speed, temperature, RH, and a dummy variable indicating sampling day; *f*_WB_(distance to WB*_i_*) is the smoothed function of the distance to the WB; *f*_BQE_(distance to BQE*_i_*) is the smoothed function of the distance to the BQE; *f_s_*(*s_i_*) is the smooth function of spatial location in the Universal Transverse Mercator (UTM; U.S. Geological Survey 2001) coordinate scheme (*s*) across all sampling locations at once, and ɛ*_i_* represents the error term. The smooth function of spatial location was included to account for any local spatial variability not captured by our distance to source terms (i.e., local sources other than the WB and BQE). All of the covariates *X_i_* were modeled linearly in our primary models. We used the shortest distance from each observation to each source as our distance measure in our primary models but modified this assumption in the sensitivity analyses. For the WB, because the bridge was elevated up to approximately 40 m above ground, an effective distance term was calculated that took this height into account. The traffic count data were aggregated to 15-min intervals and needed to be transformed to match the 1-min scale of the other covariates. This was accomplished by assuming uniform traffic volume within each 15-min interval and applying 1/15th of the traffic count to each of the individual minutes in that interval.

One of the major problems with this modeling approach is that it does not formally address all of the temporal aspects of the pollutant concentrations. This problem potentially takes two forms: short-term (minute scale) temporal autocorrelation that may contribute to violations of standard regression assumptions, and long-term (across a sampling shift) time trends that may cause difficulty in differentiating spatial from temporal trends. In order to account for autocorrelation, we created an autoregressive (AR) model. In contrast to the initial additive model (Equation 1), which assumed independent errors, the errors were taken to have an empirically determined AR correlation structure of fixed order [AR(p)], where as the order of AR(p) increases, the complexity of the correlation structure increases. The AR order and the values of the autocorrelation parameters were determined empirically by fitting AR models to the residuals from the initial model and selecting the best-fit models. We then fitted the AR model with those parameter values using the gamm function from *mgcv*.

Finally, to adjust for long-term time trends (correlation over a sampling shift) inherent in the data, and to address time with a smoothed function in addition to fixed covariates, a model was fitted for each pollutant using the gamm function from *mgcv* as follows:


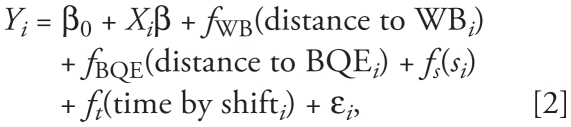


where all terms are defined as in Equation 1, but the matrix of covariates *X_i_* now includes a main effect of sampling shift, and the model includes *f_t_*(time by shift*_i_*), a smooth function of time over each sampling shift. This model also accounted for short-term temporal autocorrelation in the same manner as the AR model with the AR order and autocorrelation parameters determined from the residuals of an initial fit assuming independent errors.

We tested the sensitivity of our AR model findings to multiple statistical and parametric assumptions. Covariates that were included as linear variables were also tested as smoothed functions to see if they substantially improved model fit. For meteorological parameters, sensitivity analyses were conducted using data derived from our monitors and from the nearby LaGuardia Airport (10–11 km from the sampling zone). Additionally, we investigated the potential impact of wind direction by first stratifying the models by source (either BQE or WB) and then creating a new variable for each sampling point to determine if the monitoring was being conducted upwind or downwind of that source. Each point was assigned either an upwind demarcation (if the wind was blowing within 45° of the roadway or from the sampling location to the road) or a downwind demarcation (wind blowing from the road to the sampling location). The AR model was then rerun to see if the distance covariates differed significantly under upwind versus downwind conditions. For source strength variables, in addition to using total traffic count, we considered models that accounted for vehicle class (cars vs. trucks and buses) and vehicle speed (slow vs. medium vs. fast). We also conducted an analysis where we stratified traffic count into high and low categories using the median value and ran the AR model on each data set to see if there was any evidence of stronger distance-dependent associations under high-traffic conditions.

Another sensitivity analysis considered distance from the road in a more refined manner. We discretized the two line sources into a series of 10-m line segments, each of which was treated as an individual source. This method allows for a refined estimation of a smooth effect of distance to source within a standard mixed model, although the computational complexities meant that individual roadways could not be represented. The full details of this modeling technique can be found in Paciorek and Liu (in press). The model was specified as


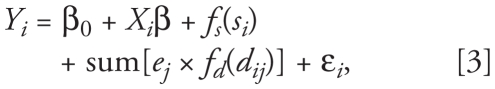


where Y*_i_*, *X_i_*β, and *f_s_*(*s_i_*) are the same as described in Equations 1 and 2, *d_ij_* is the distance from location of the measurement to the *j*th 10-m segment, and *e_j_* is the traffic estimate for that segment as a proxy for vehicular emissions.

## Results

Table 1 depicts the summary statistics for all three pollutants and other covariates. We observed significant variability in 1-min–averaged concentrations for all pollutants, with more modest variability in traffic counts. In addition, our data show very low wind speeds measured in our sampling zone, with 57% of the wind speed observations < 1 m/sec and 82% < 2 m/sec.

**Figure 2:**
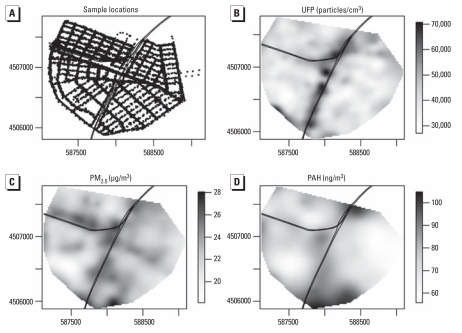
(*A*) Sampling locations. (*B*–*D*) Smoothed spatial distributions of UFPs (*B*), PM_2.5_ (*C*), and PAHs (*D*) in Williamsburg, New York. The UTM coordinates on the *x*,*y*-axes represent the location of the domain in space.

The spatially smoothed maps of concentrations demonstrate some distinct spatial patterns that differ by pollutant (Figure 2). Figure 2A depicts the location of the sampling points, illustrating the dense and relatively uniform spatial coverage. For UFPs (Figure 2B), concentrations were highest directly alongside the BQE and lower almost everywhere else. For PM_2.5_ (Figure 2C), we found the highest concentrations to the north of the WB and along varying sections of the BQE, with other localized “hot spots” and somewhat less spatial variability than for UFPs. For PAHs (Figure 2D), we found higher concentrations alongside the BQE but clustered in the northern and southern portions of our sampling zone near highway on-ramps and off-ramps.

Tables 2 and 3 show the results of the three models developed for UFPs and PM_2.5_, respectively. For PAHs, no parameters aside from the smoothed spatial surface were significant in the initial additive model, and the *R*^2^ value was very low (*R*^2^ = 0.107), so we did not consider PAHs further in our analyses. In the initial additive models for both pollutants, the smooth function of spatial location, *f_s_*(*s*), was statistically significant (Tables 2,3), and the dummy variables for sampling day were mostly significant (data not shown). After adjusting for other covariates, the smoothed spatial surface for PM_2.5_ did not display the spatial patterns observed in Figure 2. This result suggests potential overfitting in the original smooth function of spatial location that we did not adjust for other covariates. In contrast, for UFPs, the spatial surfaces appeared consistent after controlling for distance from roadway, potentially indicating local sources beyond the BQE and WB (data not shown).

**Figure 3:**
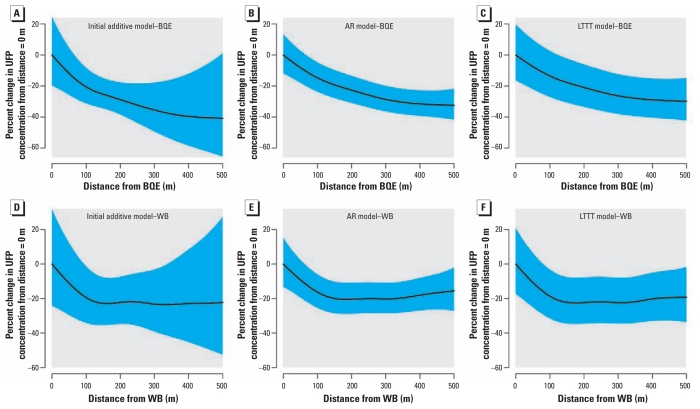
Smoothed relationship between UFP concentrations and distance from each source. (*A*–*C*) Concentrations adjacent to BQE: 50,000 particles/cm^3^ for the additive model (*A*), 42,000 particles/cm^3^ for the AR model (*B*), and 37,000 particles/cm^3^ for the long-term time trend model (*C*). (*D*–*F*) Concentrations adjacent to WB: 44,000 particles/cm^3^ for the additive model (*D*), 40,000 particles/cm^3^ for the AR model (*E*), and 36,000 particles/cm^3^ for the long-term time trend model (*F*). The shaded regions denote the 95% CI.

**Figure 4:**
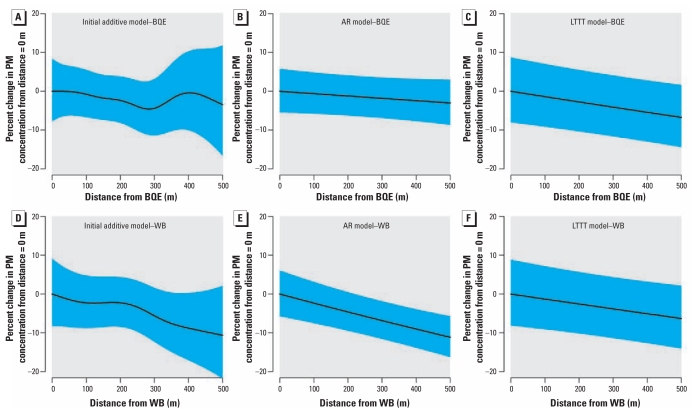
Smoothed relationship between PM_2.5_ concentrations and distance from each source. (*A*–*C*) Additive model (*A*), AR model (*B*), and long-term time trend model (*C*). (*D*–*F*) Additive model (*D*), AR model (*E*), and long-term time trend model (*F*). The shaded regions denote the 95% CI.

As hypothesized, with application of the initial additive model, UFPs demonstrated significant decreases in concentrations as a function of distance from both major roadways (Table 2, Figure 3). Predicted concentrations (adjusted for the other covariates) declined sharply within the first 100 m from both the BQE (Figure 3A) and the WB (Figure 3D), with approximately 20% decreases in concentrations associated with each roadway. For the WB, concentrations leveled off after approximately 100 m. For PM_2.5_, we observed a decrease in concentrations with increasing distance, but this association was not statistically significant (Table 3, Figure 4A,D).

When we examined other covariates in the additive model, we found that wind speed was inversely associated with both UFPs and PM_2.5_ (Tables 2,3). Ambient temperature and RH showed a significant and positive relationship with PM_2.5_ and no relationship with UFPs. Although we observed a consistent positive association between traffic counts and PM_2.5_, the traffic variables did not display this relationship for UFPs despite the clear distance-dependent relationships.

We compared our initial additive model with a more statistically robust model that included statistical terms to account for short-term temporal autocorrelation (AR model). For the UFP model, we used a first-order AR [AR(1)] correlation structure, whereas for the PM_2.5_ model we determined that a third-order AR [AR(3)] correlation structure was the appropriate fit. The major difference between the AR and initial additive models can be observed in Figure 3B and E. Within these models, the effect of proximity to the two major roadways on UFPs was similar, because both models indicated an approximate 15% decrease in concentrations within the first 100 m, with similarly shaped curves for both roadways. However, the 95% confidence intervals (CIs) were much narrower compared with the initial additive model, resulting in more precise predictions (Figure 3B,E). For the AR model, the physically interpretable parameters (i.e., defined parameters associated with emissions or atmospheric dispersion, such as traffic volume or wind speed) both for UFPs (Table 2) and for PM_2.5_ (Table 3) did not change dramatically from the initial additive model. The one exception is that for PM_2.5_ the distance terms became statistically significant (Table 3). We found an essentially linear and < 5% decrease in PM_2.5_ within 100 m of each source (Figure 4B,E).

In the models including a long-term time trend term along with an AR term, we once again used AR(1) and AR(3) for UFPs and PM_2.5_, respectively. The smooth term denoting time stratified by sampling shift and the main effect of sampling shift were generally significant (data not shown), as was the smoothed spatial surface (Tables 2,3). However, in these models, none of the physically interpretable parameters aside from distance to roadway was significant for either pollutant, and many of the effect estimates moved toward the null (Tables 2,3). For UFPs, the distance relationship was very similar to the relationship observed in the AR model (Figure 3C,F). For PM_2.5_, we still found the linear decrease in PM_2.5_ within 100 m from both sources of interest (Figure 4C,F).

We conducted a series of sensitivity analyses to test the robustness of our regression models, focusing on the AR models. First, because of the potential complexities introduced by considering both major roadways simultaneously, we reconstructed our models using only one roadway term. Model *R*^2^ and parameter estimates did not vary considerably. We tested smoothed forms for all covariates included as linear terms, with no significant improvements in model fit.

To assess the sensitivity of our results to using a different meteorological data source, we re-created our regression models with data collected from LaGuardia Airport, which exhibited much greater wind speeds (mean, 5.5 m/sec). Using the LaGuardia data, the wind speed parameter was no longer statistically significant and moved toward the null [e.g., in the UFP model, the coefficient changed from −0.046 (95% CI, −0.092 to 0.000) to −0.006 (95% CI, −0.031 to 0.019)]. For PM_2.5_, the traffic count variables were no longer significant and moved toward the null. The BQE distance variable was only nearly significant (*p* = 0.053). We found no fundamental change in the significance or interpretation of other covariates.

As mentioned above, because of a preponderance of low wind speeds, we did not include wind direction in the models. However, we tested its inclusion by evaluating the influence of upwind and downwind conditions on the smoothed distance term for both the WB and the BQE. Although we found no influence of wind direction on the concentration gradient from the WB, the association between distance and UFP concentrations for the BQE demonstrated a characteristic nonlinear decay under downwind but not upwind conditions [see Supplemental Material, Figure 1 (doi:10.1289/ehp.1002519)]. That said, the distance term remained statistically significant under upwind conditions for the BQE, reinforcing that our low wind-speed conditions reduce the influence of prevailing winds.

We also tested the impact of using alternative traffic covariates: traffic counts broken down by vehicle class (cars vs. trucks and buses) and by vehicle speed (slow vs. medium vs. fast). When we added these variables to the models in place of the total vehicle count variable, the *R*^2^ values for both UFPs and PM_2.5_ were not substantially affected. For PM_2.5_, counts of cars were a significant positive predictor of pollutant concentrations for both roadways. Truck and bus counts were either inversely associated (BQE) or not associated (WB) with PM_2.5_ concentrations. This relationship did not hold for UFPs, because neither car counts nor truck and bus counts were positively associated with pollutant concentrations. For the WB, both car and truck and bus counts were inversely associated with UFP concentrations. We found some modest evidence of a speed effect on UFPs, because fast-moving cars on the WB were positively associated with pollutant concentrations, but we did not observe this on the BQE or for PM_2.5_. We also divided the data set into observations with high versus low traffic counts and refitted the AR models, albeit with a greatly reduced sample size. For UFPs, we observed the expected, smoothed distance association from the BQE during the periods of high traffic and observed a linear association during periods of low traffic. For PM_2.5_, the BQE distance term was significant only during periods of high traffic, and the WB distance term was significant only during periods of low traffic and had a nonlinear shape. None of the traffic variables for PM_2.5_ was significantly positively associated with concentrations in either of the high- or low-traffic models.

Finally, we tested the sensitivity of our conclusions about the effects of distance on concentrations by using a discretized source model (Equation 3). The overall shape of the distance curve [see Supplemental Material, Figure 2 (doi:10.1289/ehp.1002519)] is similar to those derived previously (Figure 3), with UFP concentrations most significantly decreasing within the first 100 m. This demonstrates that our regression model conclusions are robust to consideration of more complex distance relationships.

## Discussion

Our analyses demonstrated that spatial pollutant surfaces can be characterized using mobile-monitoring protocols, with clear gradients and hot spots in and around two major roadways, especially for UFPs. We were able to quantify local source contributions relative to background concentrations across multiple model configurations. Distance from roadway was an important predictor of UFP concentrations, and we observed an approximate 15–20% decrease in concentrations within the first 100 m from both roadways for all models. A similar relationship has been observed in past studies (Kaur et al. 2005; Weijers et al. 2004; Zhu et al. 2002), although most of them did not take place in densely populated urban areas with multiple major sources or use a mobile-monitoring protocol. The mobile-monitoring protocol and corresponding statistical methods not only allowed us to capture this gradient but also produced a richer characterization of the full spatial surface through the combination of distance terms and a smoothed spatial surface.

In contrast, for PM_2.5_, we found no significant distance-dependent relationship in our initial additive model. However, once we formally accounted for short-term temporal autocorrelation, we saw a significant linear decrease in PM_2.5_ concentrations between two major roadways and points 500 m away, albeit small in magnitude relative to background concentrations. This finding suggests that PM_2.5_ gradients are present in urban settings but may be masked by the substantial background contribution, as argued previously (Zhou and Levy 2007). Recent work in New York reinforced that systematic modeling of urban background can help isolate the effect of local traffic sources, although in this case using dispersion models in urban street canyons (Jensen et al. 2009).

A primary goal of this study was not only to enhance the understanding of spatial patterns of air pollution but also to evaluate alternative analytical approaches for addressing factors that contribute to temporal and spatial variation. Disentangling these two factors is not a simple task when using mobile real-time data, and analyses that include only one of them are missing a key piece of the puzzle. To try to characterize both the temporal and spatial variability, we used varying analytical approaches each with different strengths and weaknesses. With our initial additive model (Equation 1), we accounted for the temporal variability only by using time-varying meteorological and source covariates and a dummy variable for sampling day. This model is simple to create, execute, and explain and included physically interpretable parameters, thereby maximizing its interpretability and potential generalizability. However, by neglecting the short-term temporal autocorrelation, we had very large uncertainty when looking at the predicted concentrations of UFPs with varying distance to nearest source (Figure 3A,D).

In contrast, when we used a model to account for short-term temporal autocorrelation (our AR model that incorporated a covariance structure), we had much less uncertainty about the distance parameters. The uncertainty in estimating the smooth distance effect is directly affected by the estimated residual spatial surface. If this surface is estimated to be locally heterogeneous, it is more difficult to separate residual spatial variability from the effect of distance to roadways because of concurvity, the nonlinear analogue to collinearity. In the AR model, local spatial variations are downweighted because of the estimated temporal autocorrelation between nearby measurements on a route, so the best estimate of the residual spatial surface becomes much less spatially heterogeneous than in the initial additive model, with the result that the uncertainty in the distance effect is greatly reduced. This new model is more statistically complex, but all of the physically interpretable parameters remained significant for both pollutants. This is in direct contrast to the model in which we incorporated a smooth function of time. In that model, none of the physically interpretable covariates (e.g., temperature) was statistically significant. This is likely due to concurvity between the smooth long-term time-trend terms and the time-varying covariates, with the smooth terms of time taking explanatory power away from the time-varying and physically interpretable variables. If one were interested solely in characterizing pollutant gradients from roadways, then the AR model may be the most appropriate model to use given the reduced uncertainty (compared with the initial additive model) and the increased physical interpretability (compared with the model with long-term time trends). Despite these trade-offs, the results of the three models generally agreed with one another for the distance to source parameters, reinforcing the robustness of our findings. Note that the issues involved in estimating regression coefficients in the face of temporal and spatial correlation remain open questions in the statistical literature (Houseman et al. 2006; Paciorek 2010; Reich et al. 2006).

One of the parameters that we expected would add to physical interpretability was traffic counts, which we collected in real time. However, for UFPs, we did not observe the expected relationship of increasing traffic counts leading to increasing concentrations. For PM_2.5_, in the initial additive and AR models, increasing vehicle counts were significantly associated with increasing concentrations, but not once we incorporated a smooth function of time.

Multiple factors could explain these findings. Sampling occurred only during the daytime and on weekdays, leading to a narrow range of traffic counts (Table 1). More generally, in this setting, traffic counts may not be a good proxy for vehicular emissions. For example, when traffic counts are low, it could indicate that few vehicles are present and therefore emissions are low or that there is substantial congestion and vehicles are not moving much, leading to increased emissions. We did stratify traffic counts by speed to test for this hypothesis, with some suggestive findings, but we found a lack of consistency across pollutants and roadways. In future studies, additional ways to characterize traffic emissions should be explored in addition to traffic counts, because they did not provide a complete picture of vehicle emissions.

Our study has limitations that influence the interpretability and generalizability of our findings. Sampling occurred only in the summer and during the daytime. It would have been ideal to sample over each of the four seasons, at various times of day, to fully characterize the traffic and air pollution relationship. Previous studies have shown that seasonality can affect mobile-source air pollutant concentrations (Nanzetta and Holmén 2004), and we might observe more spatial heterogeneity for PM_2.5_ in the winter given less formation and regional transport of sulfates. Our sampling period also largely involved low wind conditions, which may have affected both the observed spatial patterns and the influence of factors such as wind direction. In terms of source characterization, missing low-traffic hours as well as morning and evening rush hours limited the range of traffic conditions and may have contributed to the lack of significance for traffic predictors. We also lacked real-time characterization of traffic on surface roads in the area, although in-field observations indicated low volumes within our zone. More generally, mobile monitoring has some inherent limitations, wherein sampling in multiple seasons at all times of day in all locations simultaneously is logistically infeasible. Finally, the models detailed here are not readily applicable for out-of-sample predictions because of the addition of the purely statistical covariates, such as the smoothed spatial surface and time variables. This potentially points to the strengths of a dispersion modeling approach for characterizing small-scale spatial variability. However, our statistical methods reasonably disentangled spatial and temporal variability, and the large sample size in our study helped contribute to robust findings and improved inference about the relationships between the covariates of interest and measured pollutant concentrations.

Despite these limitations, our work provided key insights that could inform both future exposure studies and public policy decisions. In traffic-affected urban areas, siting buildings or facilities containing sensitive subpopulations more than 100 m from the nearest roadway could reduce UFP exposures by up to 20%, even given multiple proximate sources. Reflecting similar findings from prior studies, California recommendations state that new sensitive land uses should not be sited closer than 500 ft from the nearest highway (California Air Resources Board 2005); our work supports the utility of these recommendations. Furthermore, community-scale epidemiology or risk assessment studies could greatly benefit from the high-resolution exposure data we generated, and our methods could be applied in many contexts. In particular, development of spatial surfaces of UFPs would be an important first step toward better understanding public health risks and ultimately determining the necessity of future regulations.

## Conclusions

In an urban setting with complex terrain and two major roadways, we observed spatial heterogeneity of 1-min averaged concentrations of both UFPs and PM_2.5_. A mobile-monitoring protocol was able to capture spatial gradients of air pollutants, allowing spatial gradients from multiple roadways to be simultaneously characterized relative to background while accounting for physically interpretable parameters and factoring in techniques to account for autocorrelation. A variety of statistical models consistently found sharp decreases in UFP concentrations within the first 100 m from two major roadways, helping to identify exposure hot spots and characterizing spatial concentration patterns within this community.

## Figures and Tables

**Table 1:** Summary statistics for the mobile-monitoring sampling data.

Parameter	Observations (*n*)	Mean ± SD	Median	5th percentile	95th percentile
UFP concentration (particles/cm^3^)	8,225	44,000 ± 24,800	39,800	15,900	87,500
PM_2.5_ concentration (μg/m^3^)^a^	8,354	36 ± 30	29	10	129
PAH concentration (ng/m^3^)	7,453	76 ± 55	55	8	212
Traffic count per minute					
WB	9,598	13.7 ± 2.3	13.2	10.2	17.7
BQE	9,553	36.7 ± 6.5	38.4	24.5	44.9
Wind speed (m/sec)	7,913	1.3 ± 1.0	0.9	0.4	3.6
Temperature (°C)	9,441	26.3 ± 3.5	26.7	19.8	30.7
RH (%)	9,441	45.8 ± 11.3	45.4	27.3	66.4

a Measured using DustTrak, which has a known but consistent bias by a factor of 2.5–3 relative to gravimetric measurements (Chang et al. 2001).

**Table 2:** Results of the models for log-transformed 1-min–averaged UFP concentrations (*n* = 2,495).

	Initial additive model				AR model				Model with long-term time trend			
Model parameter	Parameter estimate	Lower 95% CI	Upper 95% CI	*p*- Value	Parameter estimate	Lower 95% CI	Upper 95% CI	*p*- Value	Parameter estimate	Lower 95% CI	Upper 95% CI	*p*- Value
Traffic count per minute												
WB	−0.030	−0.040	−0.020	< 0.001	−0.029	−0.043	−0.016	< 0.001	−0.012	−0.029	0.005	0.153
BQE	−0.002	−0.006	0.003	0.467	−0.001	−0.007	0.005	0.767	−0.002	−0.010	0.005	0.571
*f*_WB_(distance from WB) (m)^a^	NA	NA	NA	0.030	NA	NA	NA	0.002	NA	NA	NA	< 0.001
*f*_BQE_(distance from BQE) (m)^a^	NA	NA	NA	0.033	NA	NA	NA	< 0.001	NA	NA	NA	< 0.001
*f_s_*(*s*) (m)^b^	NA	NA	NA	0.005	NA	NA	NA	0.012	NA	NA	NA	0.063
Wind speed (m/sec)	−0.073	−0.108	−0.037	< 0.001	−0.046	−0.092	0.000	0.048	0.008	−0.044	0.060	0.767
Temperature (°C)	0.014	−0.002	0.030	0.092	0.010	−0.013	0.032	0.387	0.019	−0.006	0.045	0.140
RH (%)	0.003	−0.001	0.007	0.171	0.003	−0.003	0.009	0.281	−0.004	−0.012	0.005	0.388
*R*^2^	0.242				0.220				0.323			

CI, confidence interval; NA, not applicable. The covariates for sampling day, effect of shift, and the smooth time trends are not included.

a Results are depicted graphically in Figure 3.

b Spatial surface.

**Table 3:** Results of the models for log-transformed 1-min–averaged PM_2.5_ concentrations (*n* = 2,551).

	Initial additive model				AR model				Model with long-term time trend			
Model parameter	Parameter estimate	Lower 95% CI	Upper 95% CI	*p*-Value	Parameter estimate	Lower 95% CI	Upper 95% CI	*p*-Value	Parameter estimate	Lower 95% CI	Upper 95% CI	*p*-Value
Traffic count/minute												
WB	0.017	0.011	0.022	< 0.001	0.012	0.004	0.020	0.002	1.987E-04	−0.008	0.008	0.961
BQE	0.006	0.004	0.009	< 0.001	0.004	0.000	0.008	0.029	7.692E-05	−0.004	0.004	0.966
*f*_WB_(distance from WB) (m)^a^	NA	NA	NA	0.286	NA	NA	NA	0.005	NA	NA	NA	0.019
*f*_BQE_(distance from BQE) (m)^a^	NA	NA	NA	0.283	NA	NA	NA	0.043	NA	NA	NA	0.003
*f_s_*(*s*) (m)^b^	NA	NA	NA	< 0.001	NA	NA	NA	0.001	NA	NA	NA	< 0.001
Wind speed (m/sec)	−0.105	−0.123	−0.087	< 0.001	−0.071	−0.095	−0.047	< 0.001	−0.008	−0.032	0.017	0.550
Temperature (°C)	0.032	0.024	0.040	< 0.001	0.033	0.020	0.046	< 0.001	0.012	−0.001	0.024	0.070
RH (%)	0.014	0.012	0.016	< 0.001	0.015	0.011	0.018	< 0.001	−0.002	−0.006	0.003	0.466
*R*^2^	0.801				0.796				0.852			

CI, confidence interval; NA, not applicable. The covariates for sampling day, effect of shift, and the smooth time trends are not included.

a Results are depicted graphically in Figure 4.

b Spatial surface.
